# European Burns Association (EBA)—Summer 2024 News

**DOI:** 10.3390/ebj5020017

**Published:** 2024-06-14

**Authors:** Stian Almeland, Nadia Depetris, on behalf of the European Burns Association Executive Board

**Affiliations:** European Burns Association, 5201 AK ‘s-Hertogenbosch, The Netherlands

The European Burns Association (EBA), as the proud owner of the *European Burn Journal* (*EBJ*), is committed to fostering collaboration and maximizing the impact of our common goals, i.e., improving burn care, sharing knowledge, promoting research, and providing education toward Europe and beyond. Together, EBA and *EBJ* aim to encourage new ideas, connect professionals to better help burn patients, and set higher standards for burn treatment.

Over the past two years, the *EBJ* has made significant progress, publishing approximately 40 articles annually with a rejection rate of 34%, and the time for the first decision after submission is approximately 30 days from submission. This highlights our commitment to timely and impactful research dissemination. Our sincere appreciation goes out to our Editors-in-Chief, Professor Naiem Moiemen and Professor Peter M. Vogt, as well as the dedicated journal team at MDPI.

Looking ahead, we aim to expand the EBA’s services to members and stakeholders involved in burn injury research and care, including clinical, basic, and translational research, as well as addressing the bio-psycho-social aspects of burn injuries, prevention, epidemiology, and treatment. To this end, we are pleased to announce several Special Issues based on suggestions from the *EBJ* December 2023 Editorial Board meeting.

**We are pleased to highlight the success of the following three recent webinars:**

1. EBA webinar titled “Gender Perspectives on Burn Care” (9 March 2024).
2. *EBJ* webinar titled “Burn Injuries Associated with Wars and Disasters” (20 February 2024).
3. EBA webinar titled “Very Early Excision after Severe Burn Injuries” (5 June 2024).

These webinars are available on-demand for the EBA members.

**Furthermore, we are excited to announce the following two upcoming events:**

**1.**  **Emergency Management Severe Burns (EMSB) course in Porto, Portugal**

- Date: 16 October 2024.
- This highly interactive course focuses on the emergency assessment and stabilization of severe burns, alternating lectures, group discussions, and life-like case simulations.
- A certificate of completion is provided on successful completion of the whole course.
- Numbers are limited to a maximum of 25 participants.

**2.**  **EBA Educational Course in Porto, Portugal**

- Date: 17–18 October 2024.
- This course focuses on the relationship between burn injuries and infections, offering an opportunity for professionals to expand their knowledge and skills by engaging in multidisciplinary fruitful discussions.
- Small group workshops will focus on specific topics (ethics, surgical planning, infection control and treatment, nursing, and psychosocial issues related to infection control).
- The best selected abstracts will be published in the *EBJ*, with special awards sponsored by the publisher.
- Special registration fees are available for EBA members.

Finally, save the date for the 21st EBA Conference in Berlin, from 3–6 September 2025. Stay tuned for more details on the conference theme and agenda.

For more information about EBA activities and resources, visit our website: https://www.euroburn.org/ (accessed on 28 May 2024) and explore our EBA Burn Centers directory: https://www.euroburn.org/burn-centres/ (accessed on 28 May 2024).

We eagerly anticipate your active participation and engagement in these initiatives. Together, we can continue to advance burn care and research.

